# The utility of transcranial color Doppler in cerebral hyperperfusion syndrome

**DOI:** 10.3389/fneur.2023.1223016

**Published:** 2023-07-31

**Authors:** Marija Bender, Branko Malojčić

**Affiliations:** ^1^Department of Neurology University Hospital Mostar, Mostar, Bosnia and Herzegovina; ^2^Department of Neurology, University Hospital Center Zagreb, School of Medicine, Zagreb, Croatia

**Keywords:** cerebral hyperperfusion syndrome, transcranial Doppler, carotid endarterectomy, intracranial hemorrhage, stroke

## Introduction

Cerebral hyperperfusion syndrome (CHS) is a relatively rare but life-threatening complication following a carotid recanalization procedure. It was first described by Caplan in 1978 and still remains a matter of ongoing debate due to its vague definition, complex pathophysiology, and lack of cutoff points for defining hyperperfusion (1, 2).

CHS is traditionally considered to be a combination of clinical features with evidence of hyperperfusion and is defined as an increase in cerebral blood flow (CBF) of more than 100% over the baseline value (3).

Although this complication occurs most often after carotid revascularization procedures, CHS has been associated with other procedures as well. It is possible to develop CHS after intracranial stenting, mechanical thrombectomy, high-flow superficial temporal artery to middle cerebral artery bypass, and cardiac procedures that augment cardiac output such as heart transplantation or congenital aortic stenosis (4, 5).

The exact mechanism of this phenomenon is unclear, but it is thought that CHS is a result of impaired cerebral autoregulation. In settings of chronic low flow, due to severe carotid stenosis, cerebral autoregulation will result in maximum vasodilatation downstream. These vessels will eventually lose their ability to autoregulate and after the restoration of blood flow, it will not be able to constrict in response to systemic blood pressure increases, resulting in cerebral hyperperfusion, loss of vessel integrity, disruption of the blood–brain barrier (BBB), and eventually cerebral edema and/or intracerebral hemorrhage (ICH). Autoregulation disorder usually recovers within a few days to a few weeks, which overlaps with the occurrence of the syndrome; most cases of CHS will develop within the first few days, but delayed presentation is also possible (6).

The incidence of CHS after recanalization procedures is reported to range from 1.16 to 4.6% in previous studies. This variability in incidence between studies is most likely due to different inclusion criteria and different definitions of CHS. A retrospective meta-analysis of 13 studies, including 4,689 patients undergoing carotid endarterectomy (CEA), revealed that cerebral hyperperfusion, which is defined as an increase in flow without any clinical symptoms, hyperperfusion syndrome, and ICH occurred in 12.5, 1.9, and 0.37% of patients, respectively (7). The same author analyzed nine studies, including 4,446 patients after carotid artery stenting (CAS), and reported an incidence of CHS 1.16% and ICH 0.74%.

The clinical manifestation of CHS includes a spectrum of symptoms, ranging from mild and transient symptoms at the beginning to devastating conditions such as ICH with mortality rates as high as 50%. The typical initial presentation of CHS is a migraine-like throbbing headache, ipsilateral to a revascularized vessel, almost always associated with hypertension. If not recognized and treated in time, it can lead to seizures, confusion, and focal neurological deficit due to brain edema or intraparenchymal hematoma (5).

As mentioned above, CHS can have catastrophic consequences but it is also potentially preventable in the early stage. Multiple studies have demonstrated that rigorous blood pressure control is effective in reducing the risk and the prevalence of CHS. After CEA or CAS, CBF is linearly proportional to blood pressure, due to dis-autoregulation; therefore by lowering the blood pressure (BP), we can also reduce CBF (8–10).

Although it is quite clear that BP treatment is the mainstream in preventing CHS progression, there are no clear guidelines for BP regulation in these patients. It is considered optimal to maintain BP below 140/90 mmHg, and in a high-risk group (listed below) even below 120/80 mmHg. Few studies have shown that reducing the BP until CHS symptoms resolve, even to the level of induced hypotension, is safe and effective in eliminating the occurrence of ICH (10). Therefore, it is essential to recognize and treat these patients in time, particularly in the case of severe internal carotid artery (ICA) stenosis >90%, longstanding hypertension, poor collateral flow, recent stroke, and contralateral high-grade ICA stenosis, which are generally considered a risk factor for CHS and ICH (11).

## The role of transcranial color Doppler in diagnosing CHS

Different imaging modalities could be used to detect or screen patients for CHS. Cerebral perfusion imaging, such as single-photon emission computed tomography (SPECT), CT perfusion, or perfusion-weighted imaging, enables us to directly estimate CBF (1, 5). These methods are expensive, often unavailable, and require the application of radioisotope or contrast agents. Therefore, the lack of pretreatment CBF measurements is not unusual, which makes it impossible to assess the increase in CBF, especially if there is a contralateral carotid stenotic disease and comparison with the opposite side is not possible (1, 12).

One of the most available and most commonly used is transcranial color Doppler (TCD) (13). TCD monitoring can provide real-time information on cerebral blood flow dynamics. Changes in middle cerebral artery (MCA) velocity, measured with TCD, correlate well with changes in CBF because the diameter of MCA is not altered by autoregulation. This enables us to estimate CBF most conveniently, at the patient's bedside or in the operating room, with minimal physical burden for the patient. This method is non-invasive, easily reproducible, and suitable for repeated monitoring, with the exception of a lack of temporal bone window in 10–15% of patients (14).

The utility of TCD monitoring for the evaluation and prediction of CHS was investigated in multiple studies (Table 1). Different studies assessed CBF with TCD at different time points in relation to the recanalization procedure, to determine the test with the best predictive value.

**Table 1:** Literature review of the utility of TCD in diagnosing cerebral hyperperfusion syndrome.

**References and origin**	**Aim of the study**	**No. patients (procedure)**	**TCD parameters and timing**	**Complication rate**	**Main findings**
Jansen et al. (15) Netherlands	To analyze the incidence of ICH after CEA and to correlate this complication with TCD measurements.	233 (CEA)	MCA PSV and PI; 1 min pre-clamping and 1 min after declamping	2% developed ICH	Increase of MCA PSV and PI 175 and 100%, respectively,1 min after declamping, had PPV 100%, NPV 99%, sensitivity of 80%, and specificity of 100% for prediction of ICH after CEA
Dalman et al. (19) Netherlands	To investigate whether TCD monitoring can identify patients at risk of cerebral hyperperfusion.	688 (CEA)	MCA PSV and PI; 1 min pre-clamping and 3 min after declamping	1% developed CHS	An increase of MCA PSV or/and PI 3 min after declamping had a PPV of 11.3% for CHS.
Ogasawara et al. (12) Japan	To determine whether intraoperative TCD monitoring could be used as a reliable technique to detect cerebral hyperperfusion following CEA by comparing findings with those of brain SPECT.	60 (CEA)	MCA PSV; 1 min pre-clamping, 3 min after declamping, and at the end of the procedure	n/a	The sensitivity, specificity, and PPV of MCA PSV increases >100% immediately after declamping for detection of cerebral hyperperfusion (defined as CBF increase >100% on brain SPECT compared with preoperative value) was 100, 94, and 67%, respectively. The sensitivity and specificity of the MCA PSV increase > 100% at the end of the procedure were 100% for both parameters.
Pennekamp et al. (13) Netherlands	To determine the diagnostic value for predicting CHS by adding a TCD measurement in the early postoperative phase after CEA.	184 (CEA)	MCA PSV; within 1 week before operation, 30 s pre-clamping, 3 min after declamping, 2 h after surgery	5% patients developed CHS (including ICH)	Intraoperative MCA velocity increase >100% had PPV of 13% and NPV of 95% for the occurrence of CHS. Postoperative (within 2 h) MCA velocity increase >100% had PPV of 41% and NPV of 99% for the occurrence of CHS.
Newman et al. (18) United Kingdom	To determine whether >100% increases in MCA velocity or PI at different time points after CEA were predictive of an increased risk of ICH or stroke secondary to CHS.	1,450 (CEA)	MCA PSV and PI; pre-clamping, 1 min post-declamping, 10 min post-declamping, and 30 min post-operatively.	1.1% suffered stroke or ICH due CHS	MCA velocity increase >100% at 1, 10, and 30-min post clamping had PPV of 6.3, 8.0, and 2.7%; and NPV 98.9, 99.3, and 99.3%, respectively, for ICH or stroke secondary to CHS.
Moniche et al. (14) Spain	To validate prospectively the TCD criteria in the diagnosis of CHS after CAS.	558 (CAS)	PSV, PI, CVR in MCA; before and 24 h after CAS	3.8% developed CHS (including ICH)	An increase in MCA PSV of >100% had a sensitivity of 47.6%, specificity of 93.6%, and PPV 22.7%. With a cut-off point of a 50% of increase in MCA PSV, sensitivity improved to 66.7% with a specifity of 76.3%.
Li et al. (17) China	To identify intraoperative TCD hemodynamic predictors of CHS after CEA.	969 (CEA)	Mean MCA velocity; 1 min pre-clamping, 1 min after clamping, immediately after declamping (1–10 s), 5 min after declamping, and after suturing the skin incision	CHS 3.2% ICH 1.1%	An increase of MCA velocity immediately after declamping >110% distinguishes CHS and non-CHS patients with sensitivity of 75.9%, specificity of 88.4%, PPV of 17.8%, and NPV of 99.1%. An increase of MCA velocity 5 min after declamping >50% distinguishes CHS and non-CHS patients with sensitivity of 79.3%, specificity of 78.2%, PPV of 10.7%, and NPV of 99.1%. An increase of MCA velocity after suturing the skin incision >55% distinguishes CHS and non-CHS patients with sensitivity of 79.3%, specificity of 82.1%, PPV of 12.7%, and NPV of 99.2%.

TCD, transcranial Doppler; CEA, carotid endarterectomy; CAS, carotid artery stenting; CHS, cerebral hyperperfusion syndrome; ICH, intracranial hemorrhage; PSV, peak systolic velocity; PI, pulsatility index; MCA, middle cerebral artery; PPV, positive predictive value; NPV, negative predictive value; min, minute; s, seconds; h, hour; CBF, crebral blood flow.

At first, studies were focused on intraoperative MCA blood flow velocity measurements, usually at 2- or 3-time points (before, 1, and/or 3 min after recanalization) (15).

An increase in MCA blood flow velocity or PI >100% over the baseline values, immediately after the restoration of the flow, was considered a hemodynamic definition of cerebral hyperperfusion, and this cutoff value was used to identify high-risk patients for the development of CHS (16).

However, this cutoff point for hyperperfusion is completely arbitrary, and few researchers have recently questioned its validity for routine clinical practice. They have demonstrated that more than 50% of patients would have been underdiagnosed if this traditional definition of CHS to be used (14).

Moreover, intraoperative MCA velocity measurements yielded the lowest positive predictive value (PPV) for the detection of CHS, ranging from 8 to 18 % in different studies (16–19). This is probably because most of the patients go through transient reactive hyperemia immediately after carotid recanalization (6).

In 2015, the velocity blood pressure index (VBI) was introduced as a new prognostic parameter for CHS. This parameter combines BP and velocity changes in the perioperative phase. VBI cutoff value of 2 yielded a sensitivity of 83.3% and a PPV of 62.5% in predicting CHS development, but this has not yet been validated in a larger study (20).

Few studies investigated whether additional postoperative TCD measurements might predict CHS more accurately. These studies demonstrated that postoperative measurements (1, 2, or 24 h after the recanalization) will help to more precisely identify high-risk patients for CHS (21). One study demonstrated that the PPV of TCD measurements 1-h postoperative was more than three times higher in the prediction of CHS than intraoperative measurements. The greatest benefit of these additional TCD measurements turned out to be a high-negative predictive value (NPV). In these studies, postoperative MCA blood flow velocities increase < 100% from baseline values excluding the possibility of CHS almost completely, with an NPV of 99%, and they recommended that TCD monitoring 24 h after the recanalization should be performed to identify patients who are not at risk for CHS (21).

## Conclusion

Many open questions regarding CHS remain, but it is clear that the early recognition and treatment of CHS are crucial to prevent devastating complications such as ICH. Therefore, we need to have a high level of awareness for CHS in every patient undergoing a revascularization procedure, especially in a high-risk group. TCD is considered the most suitable imaging modality for monitoring these patients, primarily because it provides us with real-time information on CBF and can be repeated as many times as needed.

However, validated TCD protocols for the detection and prediction of CHS are still lacking, thus an individual approach to each patient is needed. Once hyperperfusion has been established, we should monitor our patients more closely and treat them appropriately, until the symptoms resolve and blood flow normalizes. We should not use strict cutoff values of CBF to establish the diagnosis of hyperperfusion because this may lead to the underdiagnosis and undertreatment of these patients. In addition, we should not try to predict CHS based on single TCD measurements. Any patient at risk of developing CHS requires an individual approach with vigilant monitoring and careful therapeutic decision-making based on MCA velocity dynamics, BP values, and clinical context.

Finally, we emphasize the need for continued research in this area, preferably through a larger multicenter prospective study that would provide a significant number of patients, give a better insight into the dynamics of these patients, and perhaps shed some new light on this complex issue.

## Author contributions

All authors listed have made a substantial, direct, and intellectual contribution to the work and approved it for publication.
